# TBHQ Attenuates Neurotoxicity Induced by Methamphetamine in the VTA through the Nrf2/HO-1 and PI3K/AKT Signaling Pathways

**DOI:** 10.1155/2020/8787156

**Published:** 2020-04-13

**Authors:** Xianyi Meng, Chenghong Zhang, Yu Guo, Ying Han, Chunyang Wang, Haiying Chu, Li Kong, Haiying Ma

**Affiliations:** Department of Histology and Embryology, College of Basic Medical Sciences, Dalian Medical University, Dalian 116044, China

## Abstract

Methamphetamine (METH) leads to nervous system toxicity. Long-term exposure to METH results in damage to dopamine neurons in the ventral tegmental area (VTA), and depression-like behavior is a clinical symptom of this toxicity. The current study was designed to investigate whether the antioxidant tertiary butylhydroquinone (TBHQ) can alleviate neurotoxicity through both antioxidative stress and antiapoptotic signaling pathways in the VTA. Rats were randomly divided into a control group, a METH-treated group (METH group), and a METH+TBHQ-treated group (METH+TBHQ group). Intraperitoneal injections of METH at a dose of 10 mg/kg were administered to the rats in the METH and METH+TBHQ groups for one week, and METH was then administered at a dose that increased by 1 mg/kg per week until the sixth week, when the daily dosage reached 15 mg/kg. The rats in the METH+TBHQ group received 12.5 mg/kg TBHQ intragastrically. Chronic exposure to METH resulted in increased immobility times in the forced swimming test (FST) and tail suspension test (TST) and led to depression-like behavior. The production of reactive oxygen species (ROS) and apoptosis levels were increased in the VTA of animals in the METH-treated group. METH downregulated Nrf2, HO-1, PI3K, and AKT, key factors of oxidative stress, and the apoptosis signaling pathway. Moreover, METH increased the caspase-3 immunocontent. These changes were reversed by treatment with the antioxidant TBHQ. The results indicate that TBHQ can enhance Nrf2-induced antioxidative stress and PI3K-induced antiapoptotic effects, which can alleviate METH-induced ROS and apoptosis, and that the crosstalk between Nrf2 and PI3K/AKT is likely the key factor involved in the protective effect of TBHQ against METH-induced chronic nervous system toxicity.

## 1. Introduction

Methamphetamine (METH) is a highly addictive drug that adversely impacts physical functions, brain functions, cognition, and social support. Dependence on this drug is difficult to treat [1, 2] because of the severity of METH withdrawal symptoms. In contrast to METH withdrawal, which is characterized by sedation and depression, chronic METH exposure is well correlated with increased depression and hyperactivity [3] due to the stimulant actions of the drug. However, stress can trigger paradoxical depression during METH withdrawal despite the fact that the stimulant effect of the drug is no longer present [4, 5]. Current evidence indicates that this neurotoxic effect of METH is due to the damage it induces in the dopaminergic (DAergic) nervous system. METH competes with dopamine (DA) uptake, stimulates DA efflux via the dopamine transporter (DAT) [6], and decreases tyrosine hydroxylase (TH) activity [7]. When administered in chronic doses, METH induces long-term deficits in striatal DAergic markers, including the DAT, TH, DA, and DA metabolites [8–11]. To some extent, the loss of DAT, TH, DA, and its metabolites is due to DAergic neuron damage and the physical loss of axons [12], which is a cause of several mental diseases and plays central roles in the predisposition of chronic METH users to the development of depression-like behavior. DA neurons project from the ventral tegmental area (VTA), which is an important part of the mesolimbic DA system [13] and a key modulator of motivated behaviors, reinforcement learning, and reward processing [14, 15]. Dysfunction of this system has been implicated in neuropsychiatric disorders such as substance abuse disorders [16, 17] and depression [18]. While METH addiction has led to intense study of the influence of VTA DA neuron damage on abuse behaviors, much less is known about the relationship between METH-induced depression-like behavior and VTA DA neuron injury.

METH-induced neurotoxicity may be related to apoptosis [18], oxidative stress (OS) [19, 20], and inflammatory changes [21]. The oxidative damage-inducing action of METH may be mediated in part by reactive oxygen species (ROS) [22]. Others have shown that exposure to METH increases the content of malondialdehyde, a product of lipid peroxidation by ROS, in brain regions of METH-exposed rats [23] and METH users [24]. Additionally, some studies have shown that METH dependence and the administration of large doses of METH [25] induce long-term changes in the brain structure, function, synaptic plasticity [26], and cell death via apoptotic and neurotoxic effects [27].

Nuclear factor erythroid 2-related factor-2 (Nrf2) is a fundamental regulator of antioxidant response element-dependent transcription and plays a significant role in the cellular adaptive response to OS [28]. Under unstressed conditions, a low level of Nrf2 is maintained by Kelch-like ECH-associated protein 1, while under OS conditions, Nrf2 is released to activate antioxidant response elements, e.g., heme oxygenase-1 (HO-1), in the nucleus [29].

Phosphatidylinositol 3-kinase (PI3K) is involved in various cellular functions, such as cell growth, proliferation, differentiation, motility, and survival, by activating protein kinase B (also known as AKT) [30]. Various reports have demonstrated that the activation of the AKT signaling pathway in different cell types is sufficient to prevent cell death induced by various apoptotic stimuli or to inhibit growth factor-induced cell survival by significantly inhibiting AKT signaling. Studies have reported that activated nuclear Nrf2, in addition to having antiapoptotic effects, further regulates several endogenous redox-regulated enzymes, such as HO-1 and glutathione cysteine ligase modulatory subunit (GCLM), via phosphorylated PI3K and phosphorylated AKT [31, 32]. Based on these findings, we hypothesize that Nrf2-PI3K is likely the key crosstalk factor linked to OS and apoptosis induced by METH.

Tertiary butylhydroquinone (TBHQ), a commonly used food antioxidant permitted by China (Health Standard GB2760.2011), is widely found in oils, biscuits, and other foods. The bodily oxidation of TBHQ can provide H+ radicals, which can stop the reaction and thus play an antioxidant role [33]. Furthermore, TBHQ induces phase II enzymes and the Nrf2 signaling pathway and shows remarkable antioxidant activity in various cell types and tissues. TBHQ was reported to reduce OS-induced injury in mice with diabetes by activating the Nrf2/ARE pathway [34] and to reduce the apoptosis of human neural stem cells and other cell types [35]. TBHQ also demonstrated the ability to repair nerve cells in the brains of mice with brain injury [36]. Therefore, in the current study, a model of chronic METH exposure was established, and TBHQ was administered. Our hypothesis was tested, and the findings indicated that chronic METH exposure can induce DA neuron damage, probably via increasing OS and apoptosis, and that these changes can be alleviated by TBHQ.

## 2. Materials and Methods

### 2.1. Chronic Methamphetamine Exposure

**Figure 1:**
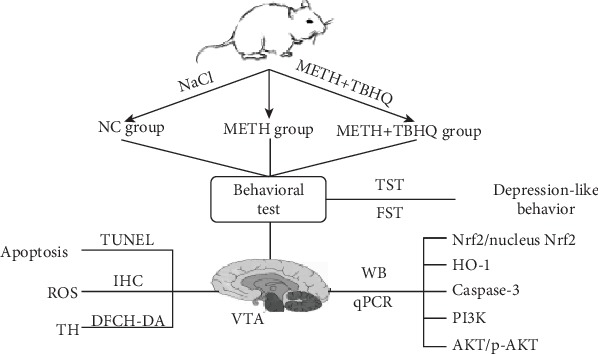
A schematic representation of protocols, treatments, behavioral tests, and biochemical analysis.

Thirty male Wistar rats (200 ± 10 g) were purchased from the Animal Resource Center of China Medical University (certificate number: Liaoning SCSK 2012-0005). All 30 rats were randomly divided into a control group, a METH-treated group (METH group), and a TBHQ administration group (METH+TBHQ group). During the first week, intraperitoneal injections of methamphetamine at a dose of 10 mg/kg were administered to the rats in the METH and METH+TBHQ groups, and METH was then administered twice per day for 6 weeks at a dose that increased by 1 mg/kg per week until the sixth week, when the daily dose reached 15 mg/kg [37, 38]. Furthermore, the rats in the control group were injected with an equal volume of a 0.9% physiological saline solution. After the administration of METH, the rats in the METH+TBHQ group then received 12.5 mg/kg TBHQ intragastrically. The rats in the control and METH groups were intragastrically administered an equal volume of 0.5% gum tragacanth. All animals were housed in a room with controlled temperature (18–22°C) and humidity (50%–70%) on an alternating 12 h light/12 h dark cycle and provided solid food and water ad libitum. All procedures were performed in accordance with the *Guide for the Care and Use of Laboratory Animals* of the National Institutes of Health (NIH), and all protocols were approved by the Institutional Animal Care and Use Committee of Dalian Medical University. A schematic representation of protocols, treatments, behavioral tests, and biochemical analysis is presented in Figure 1.

### 2.2. Forced Swimming Test (FST)

The FST was performed according to previous reports [39, 40]. The behavioral apparatus consisted of a cylindrical tank with water, and the mice could not touch the bottom of the tank or escape. The tank was made of transparent Plexiglas that was 30 cm high and 20 cm in diameter and filled with water at 22 ± 2°C to a depth of 19 cm. The mice were placed in the cylinder for 5 min, and the session was recorded. The water was replaced with clean water after each test. Three predominant behaviors were observed in the FST: immobility (when a mouse floated in the water without struggling and moved only enough to keep its head above the water), swimming (when a mouse moved horizontally in the swim cylinder, including crossing into another quadrant), and climbing (upward-directed movement of the forepaws, usually against the side of the swim cylinder) [39, 41]. Scoring was performed by an independent observer who was blinded to the treatment conditions. The total time spent engaged in each activity was analyzed.

### 2.3. Tail Suspension Test (TST)

The TST was performed according to previous reports [42]. Each mouse was individually suspended by the tail to a vertical bar. The animals were fastened by the tail for 6 min. The total duration of immobility was recorded during the last 4 min of the 6 min long testing period. The mouse was judged to be immobile when it ceased moving its limbs and body, making only those movements necessary to breathe. The immobility time was scored in real time by an independent observer who was blinded to the treatments.

### 2.4. Immunohistochemistry (IHC)

Rats were overdosed with sodium pentobarbital and transcardially perfused with 0.9% saline followed by 4% paraformaldehyde. Their brains were then extracted and postfixed in 4% paraformaldehyde in deionized water before being transferred to gradient alcohol solutions for dehydration. After dehydration, the brains were embedded in paraffin and cut into 10 *μ*m thick coronal paraffin sections. The sections were placed in an oven to dry for 2 h and stored at room temperature until IHC was performed [43].

Paraffin sections were hydrated in gradient alcohol solutions before being transferred to ethylenediaminetetraacetic acid (EDTA) for antigen repair and washed 3 times with phosphate-buffered saline (PBS). The sections were blocked with goat serum solution for 15 min at room temperature, incubated overnight at 4°C with rabbit anti-TH (1 : 200, Proteintech, USA), and washed 3 times with PBS. Then, the sections were incubated with appropriate amounts of biotin-labeled goat anti-mouse/rabbit IgG at room temperature for 20 min and washed 3 times with PBS. Subsequently, the sections were incubated with the appropriate amount of horseradish peroxidase-labeled streptavidin at room temperature for 20 min and washed 3 times with PBS. Diaminobenzidine (DAB) solution was applied to the sections for 10 s–5 min, and the sections were washed 3 times with PBS. Hematoxylin was used to stain the cell nuclei. Five random slices were selected from each group, and five randomly selected visual fields in the VTA region from each slice were observed. The mean optical density was quantified by Image-Pro Plus 5.1 software.

### 2.5. In Situ TdT-Mediated dUTP Nick End Labeling (TUNEL) Assay

The TUNEL assay was performed on tissues according to the manufacturer's instructions (TransGen Biotech, China). Briefly, deparaffinized tissue sections were washed with PBS 3 times. One hundred microliters of immunostaining permeate (0.1% Triton X-100) was added and incubated for 8–10 min at ambient temperature followed by washing with PBS for 5 min. Tissues were incubated with 50 *μ*l of a well-mixed labeling solution and 2 *μ*l of terminal deoxynucleotidyl transferase (TDT) at 37°C for 60 min in the dark to allow the tailing reaction to occur and then washed with PBS for 5 min 3 times. Then, 100 *μ*l of immunostaining permeate (0.1% Triton X-100) was added and incubated for 5 min at ambient temperature 3 times. One drop of antifade solution was added to the area containing the treated section, and the slices were mounted using glass coverslips and left to dry for 5–10 min [44]. Fluorescent cells were quantified by Image-Pro Plus 5.1 software.

### 2.6. ROS Staining and Fluorescence Microscopy Imaging

Rats were overdosed with sodium pentobarbital and transcardially perfused with 0.9% saline followed by 4% paraformaldehyde. Their brains were then extracted and postfixed for 3 h in 4% paraformaldehyde in deionized water before being transferred to 30% sucrose in deionized water. The brains were allowed to sink in the sucrose solution and were then cut on a Leica cryostat into four series of 15 *μ*m coronal sections. Frozen sections were fixed with cold acetone for 15 min at 4°C, and the serial sections were stored at -20°C until immunofluorescence (IF) analysis was performed [45].

ROS staining was performed according to the following specifications: frozen sections were washed with PBS three times for 10 min each, incubated in the probe solution (DCFH-DA) at 37°C for 30 min, washed with PBS, stained with DAPI, and sealed. The sections were then observed, and images were taken with a fluorescence microscope. The fluorescence intensity was quantified by Image-Pro Plus 5.1 software.

### 2.7. Western Blot Analysis

Nrf2, HO-1, PI3K, AKT, p-AKT, and caspase-3 were analyzed by Western blotting. Rats were anesthetized with isoflurane and immediately decapitated. The brains were quickly dissected, and sagittal sections were cut at a thickness of 30 *μ*m and stored at -80°C for Western blot experiments and reverse transcription-polymerase chain reaction (real-time PCR).

The samples were thawed, washed in ice-cold PBS, and sonicated in KeyGen lysis assay buffer (KeyGen Biotech, China). The samples were then sonicated, incubated on ice for 30 min, and centrifuged at 10, 000 × g for 20 min at 4°C. The protein concentration in the supernatant was determined by a Pierce BCA Protein Assay Kit (Life Technologies). Equal amounts of protein (20 *μ*g) were combined with loading buffer, boiled for 5 min, and loaded onto 8–12% SDS-PAGE minigels. The separated proteins were transferred onto PVDF membranes (Merck Millipore, Darmstadt, Germany). The membranes were blocked with 5% nonfat milk in TBST (0.1% Tween 20 in 20 mM Tris-HCl, pH 7.4, and 410 mM NaCl) for 2 h at room temperature and then incubated overnight at 4°C with Nrf2 (1 : 2000, Proteintech, USA), HO-1 (1 : 2000, Proteintech), PI3K (1 : 2000, Proteintech), AKT (1 : 2000, Proteintech), p-AKT (1 : 2000, Proteintech), caspase-3 (1 : 2000, Proteintech), and *β*-actin (1 : 200, Abcam, UK). The blots were washed with TBST three times for 10 min each, incubated for 1 h with horseradish peroxidase-conjugated goat anti-rabbit or goat anti-mouse IgG (1 : 5000; ZSGB-Bio, China), and washed with TBST three times for 10 min each. The bound antibodies were detected by chemiluminescence using an ECL Western blotting detection system kit (GE Amersham Biosciences, Buckinghamshire, UK) and exposed to ChemiDOC™ XRS+ Image Lab™ Software (Bio-Rad Laboratories, Inc., Hercules, CA, USA) [46].

### 2.8. Quantitative Real-Time PCR

Total RNA was isolated from tissues using a TRIzol reagent (TaKaRa, China) according to the manufacturer's instructions and treated with RNase-free DNase (TaKaRa). Single-stranded cDNA synthesis was performed using AMV Reverse Transcriptase (TaKaRa). PCR was performed using Taq DNAzyme (TaKaRa) under standard conditions (10 *μ*l of 5x PCR Buffer, 28.75 *μ*l of ddH_2_O, 0.25 *μ*l of TaKaRa Ex Taq HS, 0.5 *μ*l of each specific primer, and 10 *μ*l of cDNA) using a hot start at 94°C for 4 min; 30 cycles at 94°C for 30 s, 55°C for 30 s, and 72°C for 1 min; and a final extension at 72°C for 10 min. Glyceraldehyde 3-phosphate dehydrogenase (GAPDH) was coamplified as an internal control in each reaction. The primers for the target genes were as follows: Nrf2, forward primer 5′-GTCCAAGGAGCAATTCA-3′ and reverse primer 5′-TCGTCTTTAAGTGGCC-3′; PI3K, forward primer 5′-CCACGACGATTGCTCAA-3′and reverse primer 5′-AGCCTGCACAGGAGTAA-3′; and caspase-3, forward primer 5′-CAATGGTACCGATGTCGATG-3′ and reverse primer 5′-GACCCGTCCCTTGAATTTCT-3′.

### 2.9. Statistical Analysis

Independent sample *t*-tests were performed to detect differences between the mean values of the groups. Data are expressed as the means ± SEMs for three independent experiments and were analyzed by GraphPad Prism 5.0 software (GraphPad Software, Inc., La Jolla, CA, USA). Differences were considered significant at *p* < 0.05.

## 3. Results

### 3.1. The Antidepressant-Like Effects of TBHQ on a Model of Chronic METH Exposure

**Figure 2:**
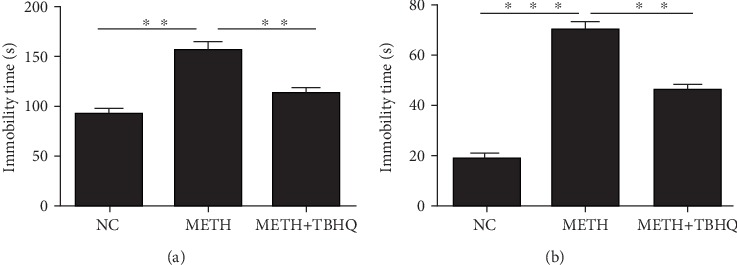
The antidepressant-like effects of TBHQ in rats treated with METH: (a) forced swimming test; (b) tail suspension test. The values represent the means ± SEMs (*n* = 5). ^∗∗^*p* < 0.01, ^∗∗∗^*p* < 0.001.

The depressive-like behaviors of METH-treated rats and METH+TBHQ-treated rats were evaluated using the FST and TST. The analysis showed that immobility times in both the FST and TST were significantly increased in the METH-treated rats compared to the control rats (*p* < 0.01 and *p* < 0.001, respectively). However, compared with the METH-treated rats, the METH+TBHQ-treated rats showed a decreased immobility time (*p* < 0.01 and *p* < 0.01, respectively) (see Figures 2(a) and 2(b)).

### 3.2. The Administration of TBHQ Rescued Neuronal Morphology in the VTA

**Figure 3:**
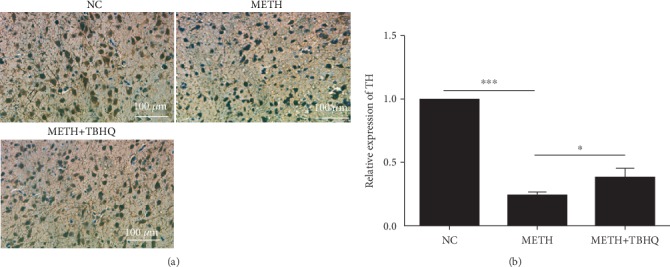
The administration of TBHQ increased the immunocontent of TH during treatment with METH. (a) Representative immunohistochemical staining for TH in the VTA. (b) TH-positive cells were quantified by the mean optical density values. The data are expressed as the means ± SEMs of three independent experiments. *n* = 5. ^∗^*p* < 0.05, ^∗∗∗^*p* < 0.001.

To verify the damage to DA neurons in the VTA induced by METH, we detected the TH protein immunocontent by immunohistochemistry. The number of TH-immunolabeled neurons was decreased in METH-treated rats compared to that in control rats (see Figure 3(a)). Quantitative analysis showed that the average optical density was significantly decreased in METH-treated rats compared with control rats (*p* < 0.001). Compared with the METH-treated rats, the METH+TBHQ-treated rats showed an increased average optical density (*p* < 0.05) (see Figure 3(b)).

### 3.3. The Antiapoptotic Effect of TBHQ in the VTA Induced by METH

**Figure 4:**
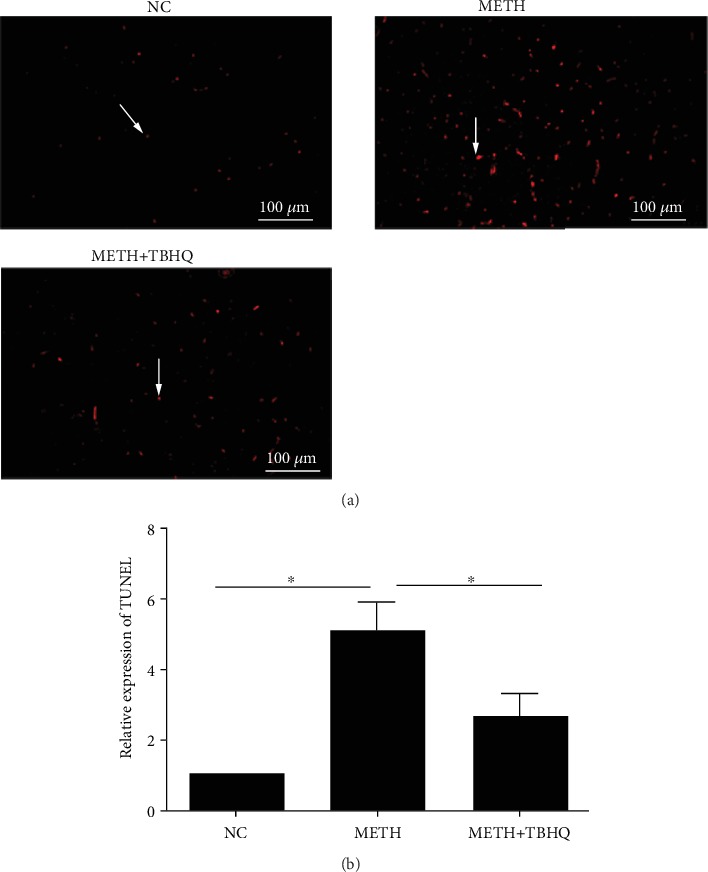
TBHQ alleviated the apoptosis induced by METH in the VTA. (a) Positive cells with a red fluorescence signal (arrows) were present in the VTA as determined by the TUNEL assay. (b) Quantitative statistical analysis of TUNEL-positive cells. A minimum of three random fields per group were used to count and calculate the percentage of positively labeled cells. The data are expressed as the means ± SEMs of three independent experiments. *n* = 5. ^∗^*p* < 0.05.

We detected the apoptosis of neurons by the TUNEL assay to further verify the damage to neurons in the VTA induced by METH. The number of positive cells with red fluorescence signals was much higher in the METH group than in the control group (see Figure 4(a)). Quantitative analysis showed that the number of positive cells was significantly increased in METH-treated rats compared with control rats (*p* < 0.05) but decreased in the VTA of METH+TBHQ-treated rats compared with METH-treated rats (*p* < 0.05) (see Figure 4(b)).

### 3.4. The Administration of TBHQ Decreased the Production of ROS in the VTA

**Figure 5:**
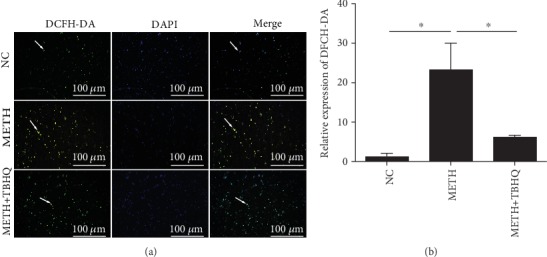
Effects of METH and TBHQ on ROS in rat VTA cells. (a) ROS production in the VTA was detected by the DCFH-DA assay. Green indicates a typical positive cell, and blue indicates a nucleus. (b) Statistical analysis of the ROS average optical density; the average optical density values were used to quantify ROS-positive cells. The data are expressed as the means ± SEMs of three independent experiments. *n* = 5. ^∗^*p* < 0.05.

Increased ROS levels are an important indicator of OS [47].  Figure 5(a) shows ROS staining in the VTA. The METH-treated rats showed stronger green fluorescence signals than the control rats. Compared to the METH-treated group, the METH+TBHQ-treated group showed distinctly less green fluorescence (*p* < 0.05) (see Figure 5(b)).

### 3.5. Effects of TBHQ on the Gene and Protein Levels of Nrf2, a Key Factor in the Antioxidant Stress Signaling Pathway, in the VTA of METH-Treated Rats

**Figure 6:**
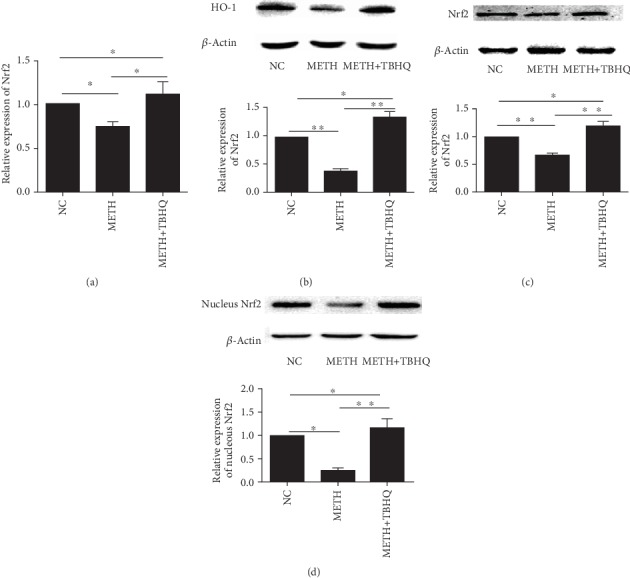
TBHQ increased the Nrf2 gene and protein immunocontent in the VAT of METH-induced rats. (a) Quantitative PCR analysis of VTA Nrf2 mRNA levels. (b) Representative Western blot images and analysis of HO-1. (c, d) Representative Western blot images and analysis of extranuclear and intranuclear Nrf2. The data are expressed as the means ± SEMs of three independent experiments. *n* = 5. ^∗^*p* < 0.05, ^∗∗^*p* < 0.01.

Changes in the immunocontent of Nrf2 partly reflected the degree of induction and development of antioxidant stress. Real-time PCR was used to detect Nrf2. The results showed that the Nrf2 gene was downregulated in the METH-treated group compared with the control group (*p* < 0.05). The Nrf2 gene in the METH+TBHQ group was upregulated compared with that in the control group and the METH-treated group (*p* < 0.05 and *p* < 0.05, respectively) (see Figure 6(a)). Western blotting was used to detect the Nrf2 protein inside and outside the nucleus. Consistent with the gene level, both the intra- and extranuclear levels of the Nrf2 protein in the VTA were significantly downregulated in the METH-treated group compared with the control group (*p* < 0.01, *p* < 0.05). In the METH+TBHQ-treated group, these levels were upregulated compared with those in the control group and METH-treated group (*p* < 0.05, *p* < 0.01, *p* < 0.05, and *p* < 0.01, respectively) (see Figure 6(c) and 6(d)).

### 3.6. TBHQ Increased the Immunocontent of HO-1 in the VTA of METH-Treated Rats

HO-1, as a downstream factor regulated by Nrf2 in the Nrf2/HO-1 signaling pathway, was detected. Compared with that in the control group, the HO-1 immunocontent in the METH-treated group was downregulated (*p* < 0.01), while in the METH+TBHQ-treated group, the HO-1 immunocontent was upregulated compared with that in the control group and METH-treated group (*p* < 0.05 and *p* < 0.01, respectively) (see Figure 6(b)).

### 3.7. Effects of TBHQ Treatment on PI3K and Caspase-3 Gene and Protein Immunocontent

**Figure 7:**
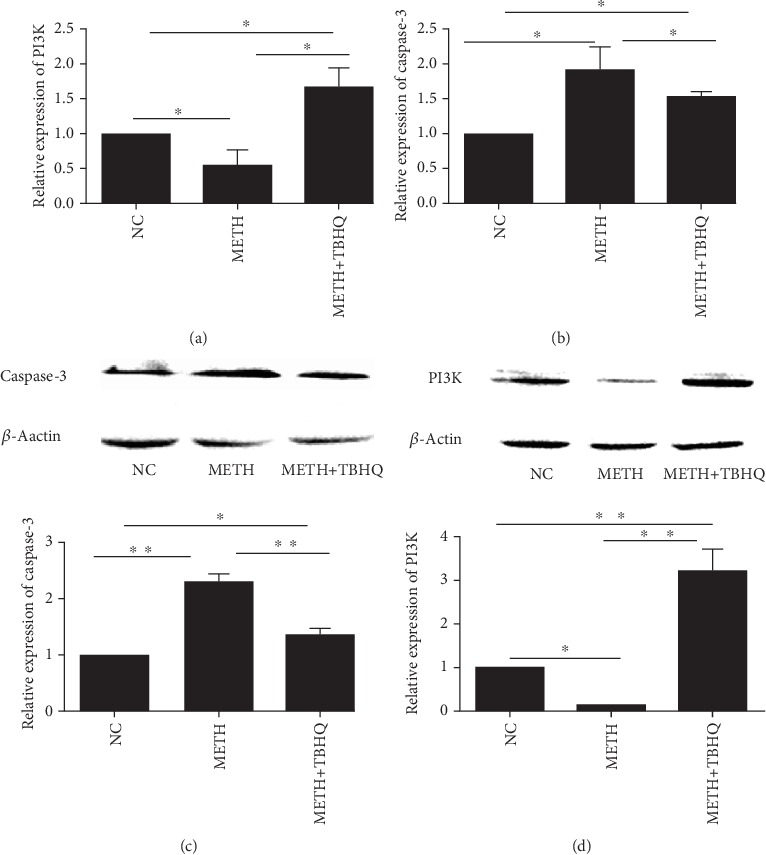
Effects of METH and TBHQ on the gene and protein immunocontent of PI3K and caspase-3. (a) Quantitative PCR analysis of VTA PI3K mRNA levels. (b) Quantitative PCR analysis of VTA caspase-3 mRNA levels. (c, d) Representative Western blot images and analysis of PI3K and caspase-3. The data are expressed as the means ± SEMs of three independent experiments. *n* = 5. ^∗^*p* < 0.05, ^∗∗^*p* < 0.01.

The mRNA levels of PI3K (see Figure 7(a)) and caspase-3 (see Figure 7(b)) were detected, revealing that PI3K was significantly decreased in METH-treated rats compared with control rats (*p* < 0.05). However, PI3K immunocontent was increased in METH+TBHQ-treated rats compared with METH-treated and control rats (*p* < 0.05 and *p* < 0.05, respectively). To further illuminate the increase in apoptosis, we assessed the mRNA levels of caspase-3, revealing that they were upregulated in METH-treated and METH+TBHQ-treated rats compared with control rats (*p* < 0.05 and *p* < 0.05, respectively). Compared with those in METH-treated rats, the mRNA levels of caspase-3 were obviously reduced in METH+TBHQ-treated rats (*p* < 0.05).

Similarly, caspase-3 protein levels were significantly increased in METH-treated rats compared with control rats, and PI3K protein levels were decreased (*p* < 0.01 and *p* < 0.05). Compared with the METH-treated rats, the METH+TBHQ-treated rats showed decreased caspase-3 protein immunocontent and increased PI3K protein immunocontent (*p* < 0.01) (see Figures 7(c) and 7(d)).

### 3.8. Effects of METH and TBHQ Treatment on AKT and p-AKT in the PI3K/AKT Signaling Pathway

**Figure 8:**
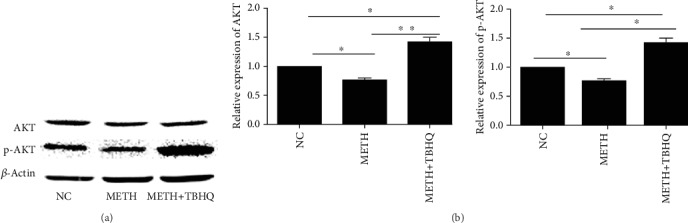
Changes in the immunocontent levels of AKT and p-AKT in the PI3K/AKT signaling pathway of METH+TBHQ-treated rats. (a) Representative Western blot images and analysis of AKT. (b) Representative Western blot images and analysis of p-AKT. Data are expressed as the means ± SEMs for three independent experiments. *n* = 5. ^∗^*p* < 0.05, ^∗∗^*p* < 0.01.

To further investigate the mechanism by which the antiapoptotic signaling pathway is involved in the effects of TBHQ, Western blot analysis was performed to detect the immunocontent of AKT and p-AKT. The results showed that the AKT (see Figure 8(a)) and p-AKT (see Figure 8(b)) immunocontent levels were significantly decreased in METH-treated rats compared with control rats (*p* < 0.05 and *p* < 0.05, respectively). However, AKT and p-AKT immunocontent was increased in METH+TBHQ-treated rats compared with METH-treated rats and control rats (*p* < 0.01, *p* < 0.05, *p* < 0.05, and *p* < 0.05, respectively).

## 4. Discussion

As a powerfully addictive drug, METH damages multiple organs, such as the brain, heart, and lungs [48–50]. This study showed that chronic exposure of Wistar rats to METH increased their immobility times in the FST and TST, which have good predictive validity and allow the rapid and economical detection of substances with potential antidepressant-like activity [51]. METH induced neurotoxicity in the VTA of rats by increasing ROS and apoptosis, thus promoting changes in the structure and function of DA neurons. METH inhibited Nrf2-mediated antioxidative stress by downregulating Nrf2 and HO-1 and further induced apoptosis by decreasing PI3K, AKT, and p-AKT expression and increasing caspase-3 immunocontent. These changes were reversed by treatment with the antioxidant TBHQ through the upregulation of the Nrf2 immunocontent. TBHQ alleviated METH-induced OS and apoptosis, possibly through the interaction between Nrf2/HO-1 and PI3K/AKT. PI3K/Nrf2 is likely the key crosstalk factor between OS and apoptosis in METH-induced chronic neurotoxicity.

In this study, we found that in the VTA, the ROS levels in Wistar rats were increased by chronic exposure to METH but reversed by TBHQ, which is in accordance with previous reports showing that OS damage in the nervous system caused by METH can be attenuated by antioxidants [52]. Redox imbalance and the generation of free radicals can lead to OS [53]. ROS include ozone (O_3_), singlet oxygen (^1^O_2_), hydrogen peroxide (H_2_O_2_), the superoxide anion radical (O_2_^−^), and the hydroxyl radical (^·^OH) [54]. Many normal cellular activities produce ROS, and physiologically, cells eliminate ROS by upregulating antioxidant proteins such as superoxide dismutase, catalase (CAT), and glutathione peroxidase (GPx) to prevent cell damage [55]. A variety of exogenous factors, such as environmental toxicants, hypoxia, hyperoxia, and stress stretching, can stimulate the body to produce excessive ROS. When ROS are not effectively removed by antioxidant enzymes, OS is induced and damages cells. Therefore, the neurotoxicity of METH may be due to excessive ROS production caused by chronic exposure. TBHQ plays an antioxidant role in METH-induced OS.

To further investigate the antioxidant mechanism of TBHQ, a novel Nrf2 activator, intra- and extranuclear Nrf2, in the VTA was detected in the model because it is proved that TBHQ possesses an oxidizable 1,4-diphenolic structure that confers its potent ability to dissociate the Keap1-Nrf2 complex [56]. Under normal conditions, Nrf2 is posttranslationally and constitutively regulated in the cytoplasm by its antagonist Keap1 through targeted ubiquitination [57]. However, upon OS, the activation of Nrf2 results in the modification of Keap1 cysteine 151 and allows Nrf2 to translocate into the cell nucleus and recruit small Maf proteins to form a heterodimer [58]. The heterodimer can bind to the antioxidant response element (ARE) and eventually transactivate a battery of antioxidant enzymes, such as NQO1 and HO-1 [59]. We found that METH significantly decreased the gene and protein immunocontent levels of Nrf2 and prevented its translocation to the nucleus, which subsequently decreased the immunocontent of HO-1. However, the decrease in Nrf2 induced by METH was markedly reversed by TBHQ treatment. It was also reported that Nrf2 deficiency exacerbates METH-induced damage to DA neurons in Nrf2 knockout (Nrf2-/-) mice, indicating the involvement of Nrf2 in the pathogenesis of METH-induced neurotoxicity [60]. TBHQ showed the ability to activate Nrf2-dependent HO-1 gene during inflammation-induced oxidative stress, which probably restores the cellular redox homeostasis thereby rendering protection against oxidative stress-mediated cell death [61]. Combining these previous studies, our results demonstrate that TBHQ may ultimately reduce the production of METH-induced ROS by activating the Nrf2/HO-1 pathway, thus playing an antioxidant role in the mouse model of METH chronic exposure.

Furthermore, we found high levels of apoptosis in the VTA of rats treated with METH. Compared with those in control rats, the number of DA neurons was reduced and morphological changes were obvious in the DA neurons of METH-treated rats. METH significantly increased the immunocontent of caspase-3. However, the changes were reversed by TBHQ treatment. The caspase family is a major player in the apoptotic process. Caspase-3 is located downstream of the caspase family cascade and is the apoptotic executive protein; it can amplify the apoptotic response and play an important role in the apoptosis family. Caspase-3 activation and high immunocontent can cause apoptosis [62]. Therefore, these results indicated that TBHQ can participate in the protection of DA neurons by inhibiting apoptosis. Based on these results together with those of the behavioral tests, we deduced that chronic METH stimulation can cause depression-like behavior in rats by increasing OS- and apoptosis-induced damage of DA neurons in the VTA. However, TBHQ attenuated METH-induced neurotoxicity in DA neurons by increasing and activating antioxidative stress and antiapoptotic abilities.

PI3K/AKT signaling is crucial for neuronal survival through the inhibition of apoptosis. PI3K is a Ser/Thr kinase and phosphatidylinositol kinase [63]. The Ser/Thr kinase activity of PI3K activates the downstream target AKT. Once AKT is activated, the biological response of AKT that controls apoptosis is simultaneously activated [64]. Research has shown that caspase-3 immunocontent can be decreased by activating the PI3K/AKT pathway to exert an antiapoptotic effect and improve neuronal damage in damaged brain regions [65]. Our data presented herein revealed that METH decreased the immunocontent of PI3K, AKT, and p-AKT and that this decrease was markedly reversed by TBHQ treatment. These results further indicate that the activation of the PI3K/AKT pathway may participate in TBHQ-mediated protection to reduce caspase-3 activation and alleviate apoptosis.

**Figure 9:**
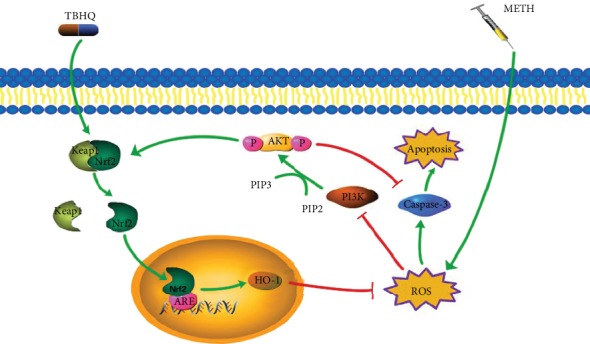
The protective role of TBHQ related to Nrf2/HO-1 and PI3K/AKT. METH chronic exposure induced the body to produce excessive ROS. On the one hand, it leads to OS disorder by reducing immunocontent of PI3K, AKT, and p-AKT; on the other hand, it promotes apoptosis through increasing caspase-3. However, TBHQ activates the Nrf2/HO-1 pathway, thereby reducing OS and protecting the normal signal transduction of the PI3K/AKT pathway to exert antioxidative stress and antiapoptotic effects.

It has been reported that PI3K/AKT signaling is an upstream pathway that regulates the nuclear translocation of Nrf2 [66]. When the body is damaged by OS caused by ROS, the phosphorylation of Ser/Thr residues is a key for Nrf2 activation, and PI3K/AKT can phosphorylate these residues [67]. Lee et al. reported that activation of the hNQO1-ARE by TBHQ is mediated by PI3K [68]. In the absence of an inducer, constitutively activated PI3K can also increase the activity of the Nrf2 target gene NQO1 and the level of glutathione [69]. In Nrf2 knockout cells, AKT shows a decreased trend of responsiveness to platelet-derived growth factor (PDGF) and/or insulin [70]. All of these studies suggest that the PI3K/AKT pathway increases the antioxidative effect of Nrf2. Therefore, we deduced that TBHQ exerts antioxidative stress and antiapoptotic effects in METH-induced DA neurons through activating Nrf2/HO-1 and regulating the PI3K-AKT pathway. Inhibitors of Nrf2 or PI3K need to be used to further verify this conclusion (see Figure 9).

## 5. Conclusions

Chronic exposure to METH causes significant damage to DA neurons in the VTA of experimental rats. The administration of TBHQ has significant protective effects against METH-induced damage based on both morphological and behavioral assessments. Our study implies that Nrf2/PI3K is likely the key crosstalk factor between OS and apoptosis in METH-induced chronic neurotoxicity. Potentially, TBHQ successfully protects DA neurons from METH-induced neurotoxicity via exerting an amplified effect on the Nrf2/HO-1 pathway, thereby reducing OS and protecting the normal signal transduction of the PI3K/AKT pathway and the antiapoptotic ability of PI3K/AKT. Concurrently, the PI3K/AKT pathway increases Nrf2 protein immunocontent and further enhances the antioxidative capacity via the Nrf2/HO-1 pathway.

## Data Availability

The data used to support the findings of this study are available from the corresponding author upon request.
